# Prevalence of COVID-19 Positive Cases Diagnosed by Real time Polymerase Chain Reaction in a Tertiary Care Hospital of Nepal

**DOI:** 10.31729/jnma.5992

**Published:** 2021-03-31

**Authors:** Khilasa Pokharel, Bhavesh Mishra, Anup Karki

**Affiliations:** 1 Department of Microbiology, Kathmandu Medical College and Teaching Hospital, Kathmandu, Nepal

**Keywords:** *asymptomatic*, *COVID-19*, *cycle threshold value*, *reverse transcriptase polymerase chain reaction*

## Abstract

**Introduction:**

The virus that causes COVID-19 is known as severe acute respiratory syndrome Coronavirus-2. This new variant of Corona Virus introduced in China has urged the massive health system resources to focus on its screening and management of sick patients worldwide. We aimed to find the prevalence of COVID-19 positive cases diagnosed by Real-time polymerase chain reaction in a tertiary care hospital of Nepal.

**Methods:**

This is a descriptive cross-sectional study that was conducted from 11th of November to 15^th^ December 2020. Nasopharyngeal and Oropharyngeal swabs were collected, and confirmation of cases of COVID-19 was done based on the detection of viral ribonucleic acid by nucleic acid amplification tests such as real-time reverse transcriptase-polymerase chain reactions. The viral genes targeted include the E, N, and ORF.

**Results:**

A total of 15247 samples have been processed, of which s (14.81%) positive cases were included in this study. There were 1427 (63.19%) male and 831 (36.68%) females. The majority of the cases were asymptomatic 1386 (61.38%). The most common age group infected was between 15 to 40 years, 841 (58.93%) male and 542 (65.22%) females. The most common presenting symptoms were cough 315 (13.95%) and fever 306 (13.55%).

**Conclusions:**

Most of the individuals reported for real-time polymerase chain reaction were asymptomatic patients who might be contagious and have the potential to transmit infection. Among symptomatic cases, common symptoms were cough and fever.

## INTRODUCTION

Coronavirus is one of the human and animal pathogens. In February 2020, the world health organization stated the disease as COVID-19, which stands for Coronavirus disease 2019.^1^ The virus that causes COVID-19 is known as severe acute respiratory syndrome Coronavirus-2 (SARS-COV-2).^2^

COVID-19 is the most contagious disease, which causes human-to-human transmission and has involved many countries.^3^ The COVID-19 can also be transmitted by an asymptomatic individual.^4^ It has been reported that infants had mild clinical manifestation and a better prognosis, and most cases were found to be asymptomatic.^5^ COVID-19 disease leads to critical care respiratory condition, which requires specialized management in the Intensive care unit in many of the cases.^6-11^ Molecular tests are the gold standard for the confirmation of COVID-19. Healthcare providers should have access to proper handwashing with soap and water or a proper supply of alcohol-based hand sanitizers.^12^

We aimed to find the prevalence of COVID-19 positive cases diagnosed by Real-time polymerase chain reaction (RT-PCR) in a tertiary care hospital in Nepal.

## METHODS

The descriptive cross-sectional study was conducted at the molecular laboratory of Kathmandu Medical College and Teaching Hospital between 11^th^ November 2020 and 15^th^ December 2020. Ethical approval was taken from the institutional review committee of Kathmandu medical college and teaching hospital (Ref no:1011202004). The sample size of this study is calculated using the formula,

$$\begin{array}{ll} \text{n} & ={\text{Z}}^{\text{2}}\times \text{p}\times \text{q}/{\text{e}}^{\text{2}}\\ & ={\left(1.96\right)}^{2}\times 0.5\times (1-0.5)/{\left(0.008\right)}^{\text{2}}\\ & =15,000 \end{array}$$

Where,

n = required sample size,
Z = 1.96 at 95% Confidence Interval
p = prevalence, 50%
q = 1-p
e = margin of error, 0.8%

Hence, a total of 15,247 samples have been performed, of which all the positive cases after performing the Real-time polymerase chain reaction (RT-PCR) test were included in this study.

Nasopharyngeal and Oropharyngeal swabs were collected and were labeled properly. Immediately after sample collection, specimens were transported to the laboratory as soon as possible to diagnose COVID-19. During the transportation of the specimen, correct handling of the specimens was done. The specimens were transported in a viral transport medium, maintaining a cold chain of 2-8°C. During transportation, repeated thawing and freezing of specimens were not done.

At present, confirmation of cases of COVID-19 was done based on the detection of viral RNA by nucleic acid amplification tests (NAAT), such as real-time reverse transcriptase-polymerase chain reactions (RT-PCR). The viral genes targeted include the E, N, and ORF to calculate the cycle threshold value (value which indicates PCR cycle number at which reaction curve intersects the threshold line) according to SARS-COV, GenBank NC_004718 (WHO 2020f).^13^

## RESULTS

Total 15247 samples have been performed, of which 2258 positive cases reported, which were categorized based on the cycle threshold value of which 1012 (44.81%) individuals were diagnosed COVID-19 positive with cycle threshold (Ct) value equal to and less than 24 725 (32.10%) individuals were diagnosed COVID-19 positive with cycle threshold value between 25 to 29 and 521 (23.07%) individuals reportedpositive with Ct value 30 and above.

**Figure 1.**
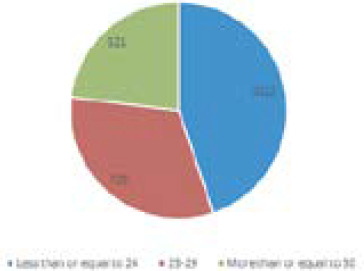
CT value distribution among COVID-19 positive patients.

Most of the cases reported were asymptomatic 1386 (61.38%), and the remaining 872 (38.61%) were symptomatic.

**Table 1:** Symptomatic and asymptomatic cases among the COVID-19 positive cases.

COVID-19 cases	n (%)
Symptomatic	872 (38.61)
Asymptomatic	1386 (61.38)
Total	2258 (100)

**Table 2:** Distribution of clinical symptoms among infected individuals.

Clinical Presentation	n (%)
Fever	306 (13.55)
Weakness	24 (1.06)
Headache	18 (0.79)
Shortness of breath	27 (1.19)
Rhinorrhea	17 (0.75)
Sore throat	22 (0.97)
Loss of taste and smell	32 (1.41)
Myalgia	95 (4.20)
Diarrhea	4 (0.17)
Cough	315 (13.95)
Nasal obstruction	3 (0.13)
Nausea-vomiting	9 (0.39)
Asymptomatic	1386 (61.38)
Total	2258 (100)

Out of a total of 2258 positive cases, 1427 (63.19%) were male, and 831 (36.68%) were female. When the male and female patients were categorized according to their ages, the most common age group infected on both genders was between 15 and 40 years.

**Figure 2.**
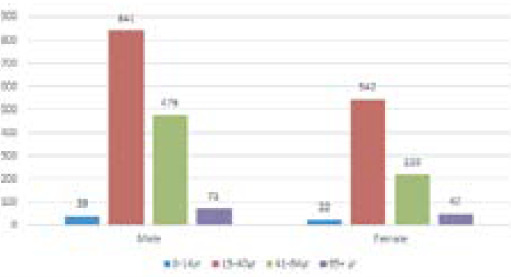
Age and gender distribution among the positive cases.

## DISCUSSION

Clinical spectrum of COVID-19 ranges from a simple cold to severe pneumonia. Laboratory test involved to identify virus was a real-time polymerase chain reaction. Individuals with COVID-19 mostly developed flu-like syndrome when respiratory samples like nasopharyngeal and oropharyngeal swabs were collected. A total of 2,258 were reported as positive for COVID-19 infection. So, on the basis of the cycle threshold value and clinical spectrum of coronavirus infection, 44.81% individuals were diagnosed to be having COVID-19 positive with cycle threshold value equal to and less than 24. Similarly, 32.10% individuals were diagnosed as COVID-19 positive with a Ct value between 25 to 29, and 23.07% individuals reported having COVID-19 infection with a Ct value of 30 above.

Out of all the cases reported positive for COVID-19 infection by performing real-time polymerase chain reaction (RT-PCR) test, 38.61% were symptomatic, and 61.38% were asymptomatic. This study is similar to the study conducted in one journal, which also reports more than half of asymptomatic cases.^16^

In this study, cough 13.95%, fever 13.55%, myalgia 4.20%, loss of taste and smell 1.41%, and shortness of breath 1.19% were found to be the major symptoms among patients suffering from COVID-19. Similar types of studies have been conducted that showed fever and cough as common symptoms among COVID-19 individuals.^17^ Centre for disease control (CDC) also listed these as common symptoms.^18^

Furthermore, out of total positive cases, 63.19% were male, and 36.68% were female. It is similar to the study conducted by Acharya et al.,^19^ which gives a strong signal toward the impact of males on COVID-19.^14,20^

Similarly, among female individuals, 2.64% were of ages between 0 to 14, 65.22% were of ages between 15 to 40, 26.47% between ages 41 to 64, and 5.65% were 65 years old and above. This is similar to other studies done where the age group between 20 to 39 years is the most infected age group among COVID-19 positive cases.^21^

The limitation of the study is that some of the cycle threshold value was in between the categories, so we followed the cycle threshold value of ORF gene on those cases. Similarly, we could not make a follow-up for the positive cases to record the recovery time.

## CONCLUSIONS

Most of the individuals who were COVID-19 positive were asymptomatic. Among symptomatic individuals, cough and fever were the most common symptoms. The common age group infected was between the age of 15 to 40 years of both male and female.
